# *Bacteroides fragilis* ameliorates *Cronobacter malonaticus* lipopolysaccharide-induced pathological injury through modulation of the intestinal microbiota

**DOI:** 10.3389/fimmu.2022.931871

**Published:** 2022-09-22

**Authors:** Na Ling, Xiyan Zhang, Stephen Forsythe, Danfeng Zhang, Yizhong Shen, Jumei Zhang, Yu Ding, Juan Wang, Qingping Wu, Yingwang Ye

**Affiliations:** ^1^ School of Food and Biological Engineering, Hefei University of Technology, Hefei, China; ^2^ Guangdong Provincial Key Laboratory of Microbial Safety and Health, State Key Laboratory of Applied Microbiology Southern China, Institute of Microbiology, Guangdong Academy of Sciences, Guangzhou, China; ^3^ Independent Researcher, Nottingham, United Kingdom

**Keywords:** *Cronobacter malonaticus*, lipopolysaccharide, *Bacteroides fragilis*, intestinal microbiota, inflammatory response

## Abstract

*Cronobacter* has attracted considerable attention due to its association with meningitis and necrotizing enterocolitis (NEC) in newborns. Generally, lipopolysaccharide (LPS) facilitates bacterial translocation along with inflammatory responses as an endotoxin; however, the pathogenicity of *Cronobacter* LPS and the strategies to alleviate the toxicity were largely unknown. In this study, inflammatory responses were stimulated by intraperitoneal injection of *Cronobacter malonaticus* LPS into Sprague–Dawley young rats. Simultaneously, *Bacteroides fragilis* NCTC9343 were continuously fed through gavage for 5 days before or after injection of *C. malonaticus* LPS to evaluate the intervention effect of *B. fragilis*. We first checked the morphological changes of the ileum and colon and the intestinal microbiota and then detected the generation of inflammatory factors, including tumor necrosis factor-alpha (TNF-α), interleukin-1 beta (IL-1β), interleukin-6 (IL-6), and interleukin-10 (IL-10) and the expression of Toll-like receptor 4 (TLR4), occludin, claudin-4, and iNOs. The results indicated that *C*. *malonaticus* LPS exacerbated intestinal infection by altering gut microbe profile, tight junction protein expression, and releasing inflammatory factors in a time- and dose-dependent manner. Intriguingly, treatment with *B. fragilis* obviously diminished the pathological injuries and expression of TLR4 caused by *C. malonaticus* LPS while increasing gut microbes like *Prevotella*-9. We note that *Shigella*, *Peptoclostridium*, and *Sutterella* might be positively related to *C*. *malonaticus* LPS infection, but *Prevotella*-9 was negatively correlated. The results suggested that the intestinal microbiota is an important target for the prevention and treatment of pathogenic injuries induced by *C. malonaticus* LPS.

## Introduction

In view of a strong epidemiological correlation between bacteremia, necrotizing enterocolitis (NEC), and meningitis infection among infants and the route of transmission (1–4), contamination of powdered infant formula caused by *Cronobacter sakazakii* has been of concern in recent years. Noteworthily, meningitis and brain abscess caused by *Cronobacter malonaticus* also occurred in a healthy full-term neonate (5). The International Commission for Microbiological Specification for Foods has ranked *Cronobacter* (formerly known as *Enterobacter sakazakii*) as a “Severe hazard for restricted populations, life-threatening or substantial chronic sequelae or long duration” (ICMSF, 2002). Therefore, the potential for diseases induced by *C. malonaticus* virulence factors should draw public attention.

Although the pathogenesis of *Cronobacter* spp. is still in its infancy now, specific virulence traits are gradually shaping up. Outer membrane proteins, including OmpA and OmpX, played vital roles in the adhesion and invasion of *C. sakazakii* to Caco-2 and INT-407 cell lines (6–9). Flagella and corresponding flagellin were involved in inflammatory responses, contributing to *Cronobacter's* attacking the host (10). Additionally, 97% of 229 *Cronobacter* species isolates possessed a homologous RepFIB plasmid encoding virulence factors such as the *eitCBAD* and *iucABCD/iutA* iron acquisition systems, as well as cpa, T6SS, and FHA that are species specific, demonstrating a strong correlation with the presence of virulence traits, plasmid type, and species (11). Our recent studies proved that LuxS, OmpA, TolB, OmpC, and LptE were the potential virulence factors (12, 13). Meanwhile, LPS is also a very important but easily underestimated component located in the outer membrane of Gram-negative bacteria (such as *Cronobacter*), which could trigger immune responses along with elevated levels of inflammatory cytokines, thereby resulting in IBD and other intestinal inflammatory disorders (14). Moreover, LPS extracted from *Escherichia coli* was reported to enhance the translocation of *Cronobacter* in the mouse model (15). Further investigation on the role of LPS in the pathogenicity of *C. malonaticus*, as well as the relationship between *Cronobacter* infections and excretive LPS, might consequently facilitate risk reduction of *Cronobacter* spp. invasion in neonates.

Currently, studies about control means for *Cronobacter* mainly focused on chemicals including sanitizers (16), natural extracts (17–19), bacteriophages (20), and prebiotic bacteria and their products (21, 22). *Bacteroides fragilis* is a representative of the *Bacteroides* genus, which harbors immune regulatory abilities (23). *B. fragilis* has the ability to reduce inflammation and can be used as therapeutics to prevent intestinal inflammatory disorders (24).

Herein, we established an inflammation model with LPS *in vivo* to investigate the potential role of *C. malonaticus* LPS in cellular pathogenicity via determining the release of inflammatory factors, expression of tight junction proteins, and tissue injuries. Furthermore, the intervention consequences of *B. fragilis* NCTC9343 in attenuation of *C. malonaticus* LPS toxicity were explored through the detection of intestinal microbiota and cellular pathogenicity.

## Materials and methods

### Animal study design

**Figure 1:**
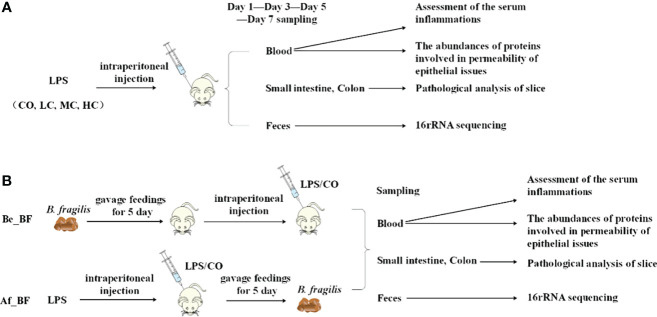
Protocol of invasion assay. **(A)** Rats (*n* = 4) were infected with LPS at densities of 0 EU/kg (CO), 2 × 10^7^ EU/kg (LC), 3 × 10^7^ EU/kg (MC), and 4 × 10^7^ EU/kg (HC), respectively. The blood, small intestine, colon, and feces were collected to assess the effects after injection of LPS for 1, 3, 5, and 7 days. **(B)** Continuous gavage feedings of *B*. *fragilis* NCTC9343 were performed for 5 days on SD young rats (*n* = 4). Be_BF, *B*. *fragilis* treatment before injection of LPS; Af_BF, *B*. *fragilis* treatment after injection of LPS.

*C. malonaticus* G361 (ST 7, O:2) was grown in Luria–Bertani (LB, Huankai, Guangzhou, China) broth at 37°C for 12–14 h, and cells were harvested for extraction of LPS. Two main sets of experiments are used: (i) LPS with densities of 2 × 107 EU/kg (LC), 3 × 107 EU/kg (MC), and 4 × 107 EU/kg (HC) were respectively used to infect Sprague–Dawley (SD) young rats (n = 4) *via* intraperitoneal injection, and (ii) continuous gavage feedings of *B. fragilis* NCTC9343 were performed for 5 days to SD young rats (n = 4) before (Be_BF group) and after (Af_BF group) the rats were infected by LPS with a density of 2 × 106 EU/ml (**Figure 1**). In the prevention group (Be_BF group), the mice were gavaged with *B. fragilis* NCTC9343 continuously for 5 days from the 10th day of birth, injected with *Cronobacter* LPS on the 15th day, and were killed 3 days later. The treatment group (Af_BF group) was injected with *Cronobacter* LPS on the 15th day and gavaged *B. fragilis* continuously for 5 days from the 16th day. The mice in the LPS, AF + LPS, and BF + LPS groups were 15-day-old when injected with *Cronobacter* LPS, and the injection amount was at a dose of 2.5 ml/kg. The blood, small intestine, colon, and feces were collected after injection of LPS for 1, 3, 5, and 7 days. Carbon dioxide was used to kill rats. The serum inflammation including TNF-α, IL-1β, IL-6, IL-10, and NO was then assessed using an ELISA kit, and the expression abundance (TLR4, occludin, claudin-4, and iNOs) was studied by quantitative PCR (qPCR, Roche LightCycler 96 system, Roche, Basel, Switzerland). The gut microbial profile was assessed by 16S rRNA sequencing in feces, and the intestinal tissues (colon and small intestine) were analyzed using H-E staining.

### Extraction of *C. malonaticus*-derived LPS

*C. malonaticus* G361 was inoculated into sterile LB broth for incubation at 37°C for 14–16 h. *C. malonaticus* cells were subsequently harvested for extraction of LPS using an LPS Extraction Kit (iNtRON Biotechnology, Inc., Seongnam, South Korea). Finally, the concentration of LPS was quantified using the Chromogenic End-point Tachypleus Amebocyte Lysate method (Chinese Horseshoe Crab Reagent Manufactory, Co. Ltd., Xiamen, China).

### Pathological analysis of slices of intestine tissue

LPS with a density of 2.0 × 107 EU/kg (LC), 3.0 × 107 EU/kg (MC), and 4.0 × 107 EU/kg (HC) were used to infect young SD rats, and normal saline was simultaneously used as control samples. The intestinal tissues (colon and ileum) were stained using H–E staining.

### Determination of inflammatory factors

Blood samples of SD young rats were collected to precipitate serum, and the inflammatory factors in serum, including NO, TNF-α, IL-1β, IL-6, and IL-10 were determined using an ELISA kit according to the manufacturer’s instruction (Yuanye, Shanghai, China). Each experiment was performed three times along with statistical analysis using SPSS 21.0 software.

### The expression of tight junction proteins and iNOs at mRNA level

The expression abundance of occludin, claudin-4, TLR4, and iNOs was determined using qPCR. The primers with detailed information are listed in **Table 1**, and GAPDH was used as a reference gene. Purified total RNA was extracted using the EZgene™ Tissue RNA Miniprep Kit (Biomiga, San Diego, CA, USA) according to the manufacturer’s protocol. The cDNA was subsequently obtained using the primerScript™ RT Master Mix (Takara, Dalian, China) according to the manufacturer’s instructions. The real-time PCR system contains 10 µl 2 × SYBR mixture, 0.4 µl for each primer (10 µM), 0.8 µl cDNA, and added RNAase-free water to 20 µl. Thermal cycling conditions were as follows: 95°C for 10 min, followed by 45 cycles of 95°C for 15 s, and 60°C for 60 s on Roche LightCycler®96 (Roche, Basel, Switzerland).

**Table 1:** Sequence of primers for quantitative real-time PCR.

Gene	Primer-forward (5’-3’)	Primer-reverse (5’-3’)
GAPDH	TGAGGTGACCGCATCTTCTTG	TGGTAACCAGGCGTCCGATA
Occludin	GCCTTTTGCTTCATCGCTTCC	AACAATGATTAAAGCAAAAG
TLR4	CAGGGAATTAGGCTCCATGA	TCCATGACAGAACGGTCAAA
Claudin-4	AAGGCCAAGGTCATGATCACAG	GAAGTCGCGGATGACGTTGT
Inos	TGGCCTCCCTCTGGAAAGA	GGTGGTCCATGATGGTCACAT

### Western blotting of TJ (tight junction) and TLR4 proteins

The procedure of Western blotting was performed as described (25) with little modification. In brief, 25 mg of small intestine tissue was lysed in RIPA buffer and measured by a microplate according to the BCA protein quantification kit (Biomiga, San Diego, CA, USA). Equal amounts of total protein were subjected to 12% SDS PAGE. Nonspecific binding sites were blocked, and the blots were incubated with appropriate primary antibodies (GAPDH, claudin-4, occludin, and TLR4) overnight. After incubation with appropriate secondary antibodies (goat anti-mouse IgG/HRP, goat anti-rabbit IgG/HRP) for 1 h at room temperature, protein bands were visualized and analyzed. The membrane was developed with PVDF membrane (0.45 μm, Immobilon) and imaged using the Chemogel imaging system (FluorChem E, Santa Clara, CA, USA). Raw densitometry data in different blots were transformed as fold change of the control mean, expressed in arbitrary units of optical density (OD), and the housekeeping GAPDH protein was used as the control.

### Intestinal microbiota analysis using 16S rRNA gene sequencing

Microbial DNA was extracted from rat fecal samples using the GD3011 Stool gDNA Miniprep Kit (Biomiga, San Diego, CA, USA). The concentrations and purities of DNA were determined by the NanoDrop 2000 UV-vis spectrophotometer (Thermo Scientific, Wilmington, DC, USA), while DNA qualities were checked by 1% agarose gel electrophoresis. The V3-V4 hypervariable regions of the bacteria 16S rRNA gene were amplified with primers 338F (5′-ACTCCTACGGGAGGCAGCAG-3′) and 806R (5′-GGACTACHVGGGTWTCTAAT-3′) by the thermocycler PCR system (GeneAmp 9700, ABI, USA). The PCR reactions were conducted using the following program: 3 min of denaturation at 95°C; 27 cycles of 30 s at 95°C, 30 s at 55°C, and 45 s at 72°C; and a final extension at 72°C for 10 min. PCR reactions were performed in triplicate using a 20-μl mixture containing 4 μl of 5 × FastPfu buffer, 2 μl of 2.5 mM dNTPs, 0.8 μl of each primer (5 μM), 0.4 μl of FastPfu Polymerase and 10 ng of template DNA. The PCR products were further purified using the AxyPrep DNA Gel Extraction Kit (Axygen Biosciences, Union City, CA, USA) and quantified using QuantiFluor™-ST (Promega, Madison, WI, USA) according to the manufacturer’s protocol.

### Bioinformatics and statistical analysis

Purified amplicons were pooled in equimolar and paired-end sequenced (2 × 300) on an Illumina MiSeq platform (Illumina, San Diego, USA) according to the standard protocols by Majorbio Bio-Pharm Technology Co. Ltd. (Shanghai, China). The raw fastq files were demultiplexed, quality filtered by Trimmomatic, and merged by FLASH with the following criteria: (i) The reads were truncated at any site receiving an average quality score <20 over a 50-bp sliding window. (ii) Primers were exactly matched allowing two-nucleotide mismatching, and reads containing ambiguous bases were removed. (iii) Sequences with an overlap longer than 10 bp were merged according to their overlap sequence. Operational taxonomic units (OTUs) were clustered with a 97% similarity cutoff using UPARSE (version7.1 http://drive5.com/uparse/) and chimeric sequences were identified and removed using UCHIME. The taxonomy of each 16S rRNA gene sequence was analyzed by the RDP Classifier algorithm (http://rdp.cme.msu.edu/) against the Silva (SSU128) 16S rRNA database using a confidence threshold of 70%.

The high-quality reads were clustered into OTUs using Mothur (26). The OTUs were used for alpha diversity within a community and were mainly measured by Chao1 and Shannon diversity indexes. Principal coordinate analysis (PCoA) was performed according to the distance matrices created by Mothur, and three-dimensional graphical outputs were drawn using SigmaPlot (version 12.0; Systat Software Inc., CA, USA). The data were filtered using QIIME software for linear discriminant analysis (LDA) of LDA effect size (LEfSe) to find a notable difference in taxonomic abundance among different treated groups. LEfSe used the Kruskal–Wallis rank-sum test with a normalized relative abundance matrix to detect features with remarkably different abundance between assigned taxa based on an alpha significance level of less than 0.05 and an effect-size threshold of 3.5 (LDA scores).

## Results

### Pathological analysis of intestine tissues treated by *C. malonaticus* LPS

**Figure 2:**
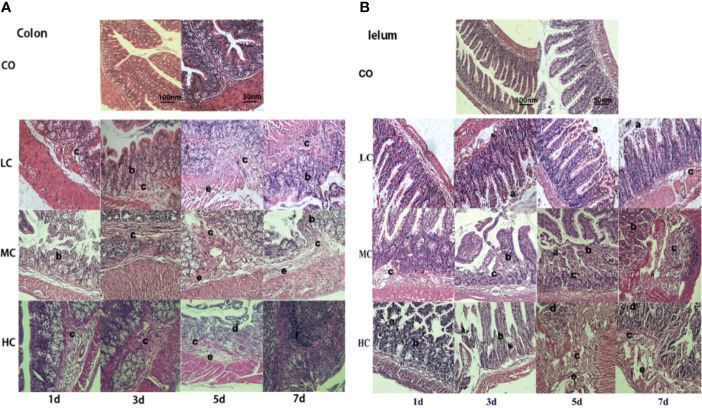
Morphological injuries of issues (**A**-Colon, **B**-Iulem) on different days after intraperitoneal injection of *c*. *malonaticus* LPS into SD rats. CO, control; LC, low concentration; MC, moderate concentration; HC, high concentration. **A**-Colon and **B**-Iulem: slight damage to villus tips **(a)**, distension and separation of lamina propria **(b)**, inflammatory cytokine infiltrates **(c)**, intestinal structure disorder **(d)**, edema in mucosa layers and submucosa **(e)**, intestinal structure seriously disturbed, and high-grade inflammatory cytokine infiltrates **(f)**.

The changes in histological morphology of the gut from rats exposed to *C. malonaticus* LPS treatment were observed. All ileum and colon samples taken from each rat pup showed marked inflammatory cytokine infiltrates, intestinal structure disorder, mucosa layers, submucosa edema, and intestinal structure disturbed seriously in LPS-treated pups compared to those from the untreated pups (**Figure 2**). Additionally, LPS caused dose- and time-dependent pathological damage in ileal and colon cells.

### Release of inflammatory factors induced by *C. malonaticus* LPS

**Figure 3:**
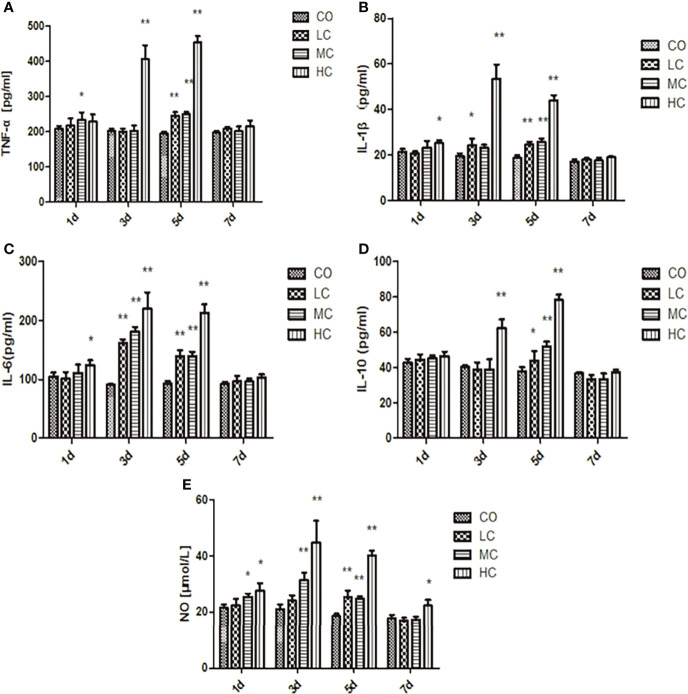
The release of inflammatory factors (**A**, TNF-α; **B**, IL-1β; **C**, IL-6; **D**, IL-10; and **E**, NO) in the serum of SD rats after intraperitoneal injection of *C. malonation* LPS for 1, 3, 5, and 7 days. ^*^ *p* < 0.05 compared to control (CO); ^**^ *p* < 0.001 compared to CO. LC, low concentration; MC, moderate concentration; HC, high concentration; TNF-α, tumor necrosis factor-α; IL-1β, interleukin-1β; IL-10, interleukin-10; IL-6, interleukin-6; NO, nitric oxide.

Inflammatory factors released from the three treated groups with low, medium, and high concentrations of LPS were both dose-dependent and time-dependent. As shown in **Figure 3**, TNF-α and IL-1β significantly increased in all groups treated with different concentrations of LPS (LC, MC, and HC group) only after treating for 5 days in comparison to the control group. While IL-6 from rat serum was markedly elevated in the HC group after the treatment for 1 day (p < 0.05), which represented a time-dependent increase (p < 0.01). Similarly, NO and IL-10 were obviously released in all LPS-treated groups after 3 days. Surprisingly, the levels of NO, TNF-α, IL-1β, IL-6, and IL-10 in serum from all groups returned almost entirely to those of the control group, except for NO in HC-treated group which was remarkably increased (p < 0.05).

### Expression of occludin, claudin-4, TLR4, and iNOs induced by *C. malonaticus* LPS

**Figure 4:**
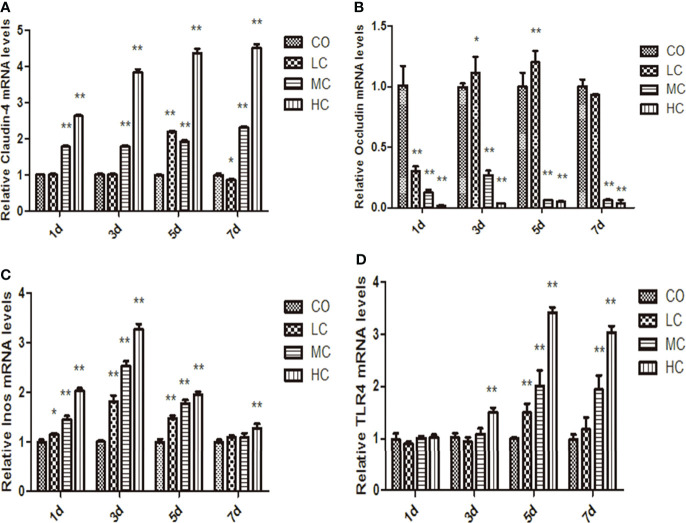
The mRNA levels of claudin-4, occludin, iNos, and TLR4 in the small intestine at different times after intraperitoneal injection of different dose of *C. malonation* LPS for 1, 3, 5, and 7 days. **(A)** claudin-4; **(B)** occludin; **(C)** iNos; **(D)** TLR4; *p < 0.05 compared to control (CO); **p < 0.001 compared to CO. LC, low concentration; MC, moderate concentration; HC, high concentration; iNOS, nitric oxide synthase; TLR4, Toll-like receptor 4.

The epithelial permeability levels were characterized by the expressions of proteins, including occludin, claudin-4, TLR4, and iNOs, which are shown in **Figure 4**. The abundance of claudin-4 was significantly increased in MC- and HC-treated groups, whereas occludin levels were decreased in the MC and HC groups (p < 0.01). Occludin in the LC group displayed the tendency to decline on day 1 but increase on days 3 and 5 compared with the control group. The expression of iNOs in LPS-treated groups within 5 days was markedly increased compared with the control group. Meanwhile, treatment with different concentrations of LPS obviously facilitated TLR4 expression, which is shown as HC (p < 0.01, days 3, 5, and 7), MC (p < 0.01, days 5 and 7), and LC (p < 0.01, day 5).

### Attenuation of *B. fragilis* on pathological injury

**Figure 5:**
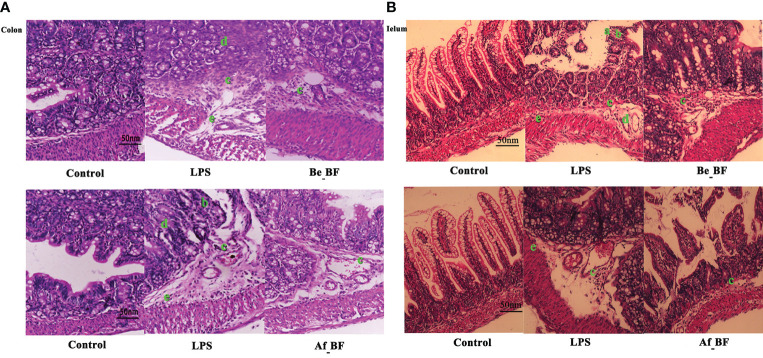
The effect of *B*. *fragilis* on the prevention and treatment of rat colon **(A)** and ileum **(B)** induced by *C*. *malonaticus* LPS. **(A, B)** Slight damage to villus tips **(A)**; distension and separation of lamina propria **(B)**; inflammatory cytokine infiltrates **(C)**; intestinal structure disorder **(D)**; edema in mucosa layers and submucosa **(E)**. LPS, LPS-treated group; Be_BF, *B*. *fragilis* treatment before injection of LPS; Af_BF, *B*. *fragilis* treatment after injection of LPS.

We further examined whether *B. fragilis* NCTC9343 influenced the gut barrier function. The results declared that injuries of colon and ileum issues caused by *C. malonaticus* LPS should be attenuated in the Be_BF group, which was with the adjunction of *B. fragilis* pretreatment compared with the group exposed to LPS alone (**Figure 5**). Likewise, *B. fragilis* posttreatment after *C. malonaticus* LPS invasion (Af_BF group) also contributed to the recovery of morphological injuries in the ileum and colon issues.

**Figure 6:**
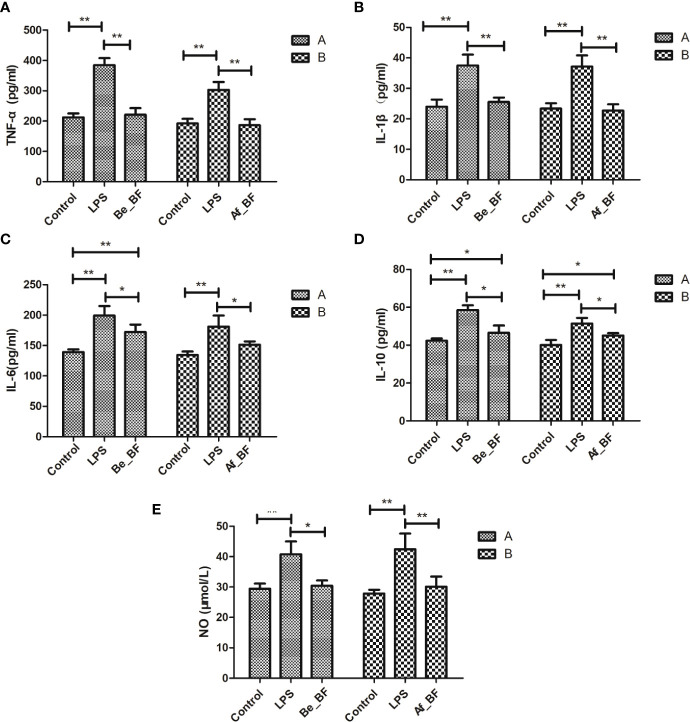
Effects of *B*. *fragilis* on the release of inflammatory cytokine levels in different groups. **(A)** TNF-α, tumor necrosis factor; **(B)** IL-1β, interleukin-1β; **(C)** IL-6, interleukin-6; **(D)** IL-10, interleukin-10; **(E)**, nitric oxide; LPS, LPS-treated group; Be_BF, *B*. *fragilis* treatment before injection of LPS; Af_BF, *B*. *fragilis* treatment after injection of LPS. *p < 0.05; **p < 0.001.

*C. malonaticus*-LPS injection obviously stimulated the level of TNF-α, IL-1β, IL-10, IL-6, and NO, while these factors were markedly reduced in both the Af_BF group and Be_BF group compared with LPS-treated group (**Figure 6**). In addition, insignificant differences for TNF-α, IL-1β, and NO were observed between *B. fragilis*–treated groups (Be_BF and Af_BF), and IL-10 in the Af_BF and Be_BF groups were notably increased (p < 0.05) compared with the control group.

**Figure 7:**
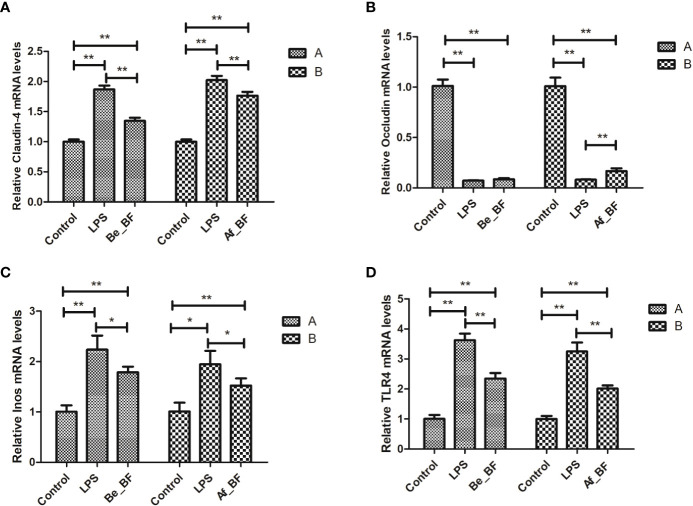
Relative expression of the release of inflammatory factors (**A**, claudin-4; **B**, Occludin; **C**, Inos; **D**, TLR4) at mRNA level in the small intestine after *B*. *fragilis* treatment. LPS, LPS-treated group; Be_BF, *B*. *fragilis* treatment before injection of LPS; Af_BF, *B*. *fragilis* treatment after injection of LPS; TLR4, Toll-like receptor 4. GAPDH was used as a reference gene; *p < 0.05; **p < 0.001.

**Figure 8:**
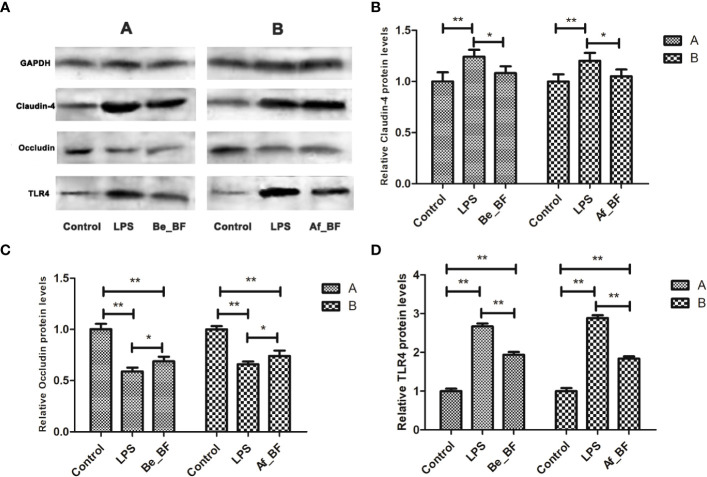
Relative expression of tight junction-related proteins and TLR4 at a proteomic level in the small intestine after *B*. *fragilis* treatment. **(A)** western blotting of TJ and TLR4 proteins; **(B)** relative Claudin-4 protein levels; **(C)** relative Occludin protein levels; **(D)** relative TLR4 protein levels; LPS, LPS-treated group; Be_BF, *B*. *fragilis* treatment before injection of LPS; Af_BF, *B*. *fragilis* treatment after injection of LPS; GAPDH was used as a reference protein; *p < 0.05; **p < 0.001.

Claudin-4, TLR4, and iNOs were adopted as the indicators of the epithelial permeability in the Af_BF-, Be_BF-, and LPS-treated groups, which markedly increased compared with those in the control group (**Figure 7**). Pretreatment and posttreatment of *B. fragilis* inhibited *C. malonaticus* LPS-induced enhancement in mRNA expression of claudin-4, TLR4, and iNOs, in which claudin-4 expression from the Af_BF group and Be_BF groups exhibited a more considerable reduction compared with the LPS-treated groups. The changes in protein expression of claudin-4, TLR4, and occludin were similar to those in mRNA (**Figure 8**).

### Alteration of gut microbiota composition by *B. fragilis* intervention

**Figure 9:**
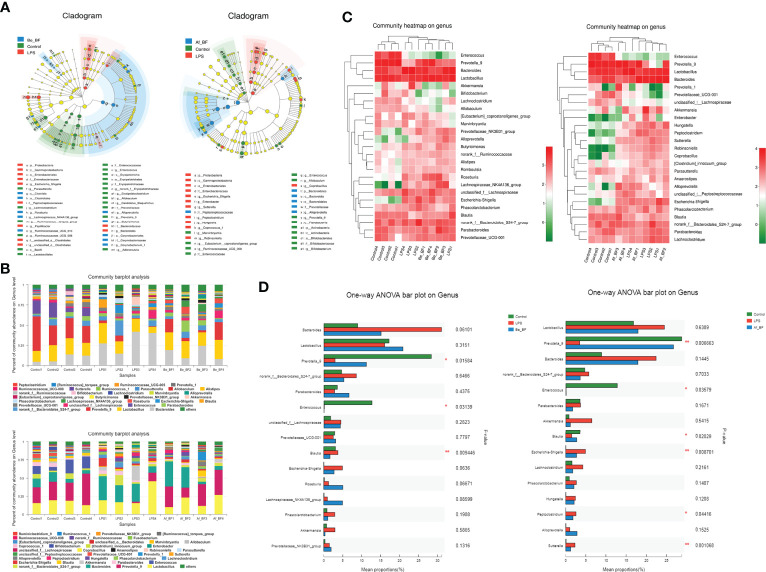
Comparison of gut microbiome composition between LPS-treated samples and *B*. *fragilis* treatment groups. **(A)** Linear discriminant analysis (LDA) coupled with effect size measurements identifies the most differentially abundant taxons among the control, *B. fragilis* treatment groups (A-1 for Be_BF group; A-2 for Af_BF group), and LPS-treated group. Enriched Taxa are shown with different colors (Control group for blue, *B*. *fragilis* treatment groups for green, and LPS-treated group for red). Only taxa meeting an LDA significant threshold of >3.5 are shown. **(B)** Community abundance ratio on the genus (B-1 for Be_BF group; B-2 for Af_BF group). **(C)** Community heatmap on genus with top 25 differential abundance (C-1 for Be_BF group; C-2 for Af_BF group). **(D)** One-way ANOVA bar plot on genus with top 15 differential abundance (D-1 for Be_BF group; D-2 for Af_BF group). *p < 0.05; **p < 0.001.

16S rDNA sequences were analyzed using the LEfSe method to determine the significant differences between organisms and clades across treatment groups. LEfSe provides three main outputs (**Figure 9A**), which describe the sizes of the effects of differences observed among Be_BF-, Af_BF-, and LPS-treated groups. LEfSe detected 18, 10, and 11 bacterial clades in control, Be_BF-treated, and LPS-treated groups, respectively. Likewise, 13, 12, and 6 bacterial clades in the control, Af_BF-treated, and LPS-treated groups were divided, respectively. These bacterial clades had relatively high abundance and showed statistically significant and biologically consistent differences. Community abundance ratio and heatmap (**Figures 9B, C**) combined with one-way ANOVA analysis (**Figure 9D**) about Be_BF-, Af_BF-, and LPS-treated groups on genus showed the distinctive patterns of enrichment-related to different groups. We found that a higher abundance of opportunistic pathogens including *Shigella*, *Peptoclostridium*, and *Sutterella* were detected when rats were injected with LPS without the intervention of *B. fragilis*, which could be distinguished from the effect of *C. malonaticus*-LPS. Additionally, *Prevotella*-9 was remarkably reduced when rats were injected with LPS and subsequently recovered to the normal after pretreatment or posttreatment of *B. fragilis*.

## Discussion

*Cronobacter* is an important foodborne pathogen associated with NEC, neonatal meningitis, and bacteremia. LPS is widely acknowledged to trigger an immune response resulting in elevated levels of inflammatory cytokines (27). Only slight or no responses occur in a host exposed to a low dose of LPS, which is considered an adaptive response defined as LPS tolerance (hyporesponsiveness) whereas the body will have an immune response and inflammation when the dose of bacterial endotoxin is high. Townsend etal. (15) demonstrated that *Escherichia coli* LPS combined with *C. sakazakii* cells facilitated the translocation of *Cronobacter* cells in rats, and the colon of neonatal rats after LPS treatment showed areas of edema, flattening, and vacuolation. However, the host responses were still unknown when the host was infected by *C. malonaticus* LPS alone. Herein, we further explored whether the diverse doses of LPS would have different effects on toxicity responses. We found that *C. malonaticus* LPS induced time-dependent and dose-dependent release of inflammatory factors, which included TNF-α, IL-6, NO, IL-10, and IL-1β in the rat model. Intriguingly, these inflammatory factors were restored to normal levels except NO after LPS treatment for 7 days, illustrating that 7 days might be a repair cycle of the host during resisting against *C. malonaticus* LPS. LPS usually induced tissue injury and significantly enhanced the production of TNF-α and IL-6 (28–30), and LPS and IL-1β acted synergistically on cytokine production by upregulating MyD88 expression in human gingival fibroblasts (31). Moreover, IL-10, which is one of the most potent anti-inflammatory cytokines in the immune system, was required for protection against inflammation in animal models (32–34). Nitric oxide plays a critical role in mediating macrophage cytotoxicity against a variety of microorganisms (35, 36). The excess production of NO also induced the release of TNF-α and IL-Iβ, which in turn triggered the host to produce an inflammatory response. In this study, the expression of iNOS was in accordance with the level of NO concentrations in the serum of rats, which was supported by previous studies (37, 38).

Previous studies have confirmed that TLR4 plays an important role in a broad range of intestinal diseases such as inflammatory bowel disease and NEC (39–41). Enterocyte TLR4 mediated phagocytosis and translocation of Gram-negative bacteria by enterocytes (42). Moreover, TLR4, as the major receptor for LPS, triggered a cascade of inflammatory responses, leading to the activation of the NF-kB signal to induce the production of proinflammatory and inflammatory factors (43). In this study, the expression level of TLR4 at mRNA level was increased in the HC group after treatment for 3–7 days. The excess level of claudin can induce tight junction fibril formation in fibroblasts cells. The level of claudin-4 was markedly increased in the *C. malonaticus* LPS-treated group compared with the control group, which reveals that the disruption of tight junctions during infection of *C. malonaticus* LPS may also trigger upexpression of claudin-4 as a host’s stress response. Correspondingly, the level of ccluding protein in the LPS-treated group was lower than that of the control group, in agreement with 44, 45). TNF-α and IL-1β were also involved in the regulation of ccluding protein (46), while IL-1β could inhibit ccluding protein by degrading I-kB and activating the NF-kB pathway (47). Therefore, inflammatory factors play a critical role in the poverty of TJ and the permeability of intestinal barriers, which are also accompanied by tissue damage. We speculated that *C. malonaticus* LPS firstly induced the expression of TLR4, and consequently triggered the release of inflammatory cytokines (TNF-α, IL-Iβ, IL-6, IL-10, and iNOS), which disrupted the TJ of intestinal epithelial cells (48, 49). Our previous results indicated that *C. malonaticus* infection caused the flattening and vacuolation of the mucosal layer (12). *C. malonaticus* LPS similarly caused morphological damages in the ileum and colon, which included inflammatory cytokine permeation, structure disorder, mucosa layer injury, and submucosa edema. The morphological injuries of the ileum and colon were more predominant in the high concentration group, and the injuries exhibited a dose-dependent tendency after treating for 3–5 days. The low dose of LPS did not cause the abnormal level of inflammatory factors, while expression of inflammatory factors obviously increased when LPS concentration was higher than that in the MC group (3 × 107 EU/kg). Although the inflammatory factors were restored to normal levels on the 7th day, the morphological injuries of colon and ileum issues were also visible. Concentrations above 2 × 107 EU/kg of *C. malonaticus* LPS could cause a distinct change in TJ proteins, although this change did not recover at day 7, as inflammatory factors did.

Probiotics have been suggested to be associated with reduced NEC incidence and severity in both animals (50–53) and infants suffering from NEC (54–56). Fan etal. (57) demonstrated that *B. fragilis* ZY-312 inhibited the deleterious effects of *C. sakazakii* in a neonatal rat model. In this study, *B. fragilis* NCTC9343 treatment obviously alleviated the morphological injuries induced by *C. malonaticus*-LPS in the ileum and colon issues. The abundance of opportunistic foodborne pathogens was higher in the LPS-treated group than those in both the control and *B. fragilis*-treated groups, and gut-microbiome similarity between CO- and *B. fragilis*-treated groups was higher compared with that between CO- and LPS-treated groups, showing that *B. fragilis* treatment is beneficial to the recovery of gut microbiome altered by LPS. Mazmanian etal. (58) found that *B. fragilis* protected the host from inflammatory disease caused by *Helicobacter hepaticus* in an animal model of experimental colitis, and a polysaccharide A produced by *B. fragilis* in the gut can ameliorate inflammatory disease in animals. Moreover, probiotics could also regulate intestinal TJ proteins to relieve intestinal mucosal permeability (59, 60). Here, weakened pathogenic injuries and lower levels of serum inflammatory factors were distinctly detected when the LPS-treated mice were co-treated with *B. fragilis*. Judging by the expression of inflammatory factors such as IL-6 and IL-10, pretreatment of *B. fragilis* plays a more effective role in attenuation of proinflammatory responses than postaddition of *B. fragilis*.

As shown in **Figure 8**, the flora structure of the AF and BE groups changed greatly. *Prevotella* is one of the three dominant bacteria in the human intestinal tract, and in its roles in health and sickness are controversial. Emerging studies found a correlation between the increased abundance of *Prevotella* at mucosal sites and disease, which includes periodontitis, bacterial vaginosis, rheumatoid arthritis, metabolic disturbance, and low-grade systemic inflammation (61). However, *Prevotella*-9 abundance was prominently reduced when the host was exposed to LPS and subsequently recovered to normal with pretreatment or posttreatment of *B. fragilis* in this study. Certainly, more effective restoration was detected in the *B. fragilis* posttreatment group. Recently, Rolhion etal. (62) reported that a Listeria monocytogenes bacteriocin limiting Listeria intestinal colonization could target *P. copri* and modulate intestinal infection. Therefore, whether *Prevotella*-9 is negatively correlated with *C. malonaticus* LPS infection is worth investigating. Moreover, *Shigella* is rarely present in normal mice but is notably enriched after LPS injection and reduced after *B. fragilis* intervention. A similar abundance trend of bacteria was also observed in *Peptoclostridium* and *Sutterella. Shigella*, which causes around 125 million diarrheal episodes per year and kills approximately 160  thousand people (63), has been identified as a major contributor to the global diarrhea burden. *Shigella* invasion could result in the destruction of the large intestinal epithelium (64). The genus *Peptoclostridium* in the family *Peptostreptococcaceae*, including *P. difficile*, is a major pathogen causing diseases ranging from antibiotic-associated diarrhea to life-threatening pseudomembranous colitis. *Sutterella* was linked to Crohn’s disease and ulcerative colitis (65) and was also a major component in a large proportion of children with autism and gastrointestinal dysfunction (66). Therefore, *Shigella, Peptoclostridium*, and *Sutterella* might be the biomarker for infection of *C. malonaticus*-LPS. Surprisingly, *Akkermansia* and *Lactobacillus* were significantly enriched within the *C. malonaticus* LPS group without the addition of exogenous probiotics, despite the fact that the genus *Akkermansia* and *Lactobacillus* have been suggested as biomarkers for a healthy intestine due to their abundance in healthy mucosa and the inverse correlation between the two genus and several intestinal disorders (67, 68). Nevertheless, (69) revealed that a high-sugar diet would induce an increased abundance of *A. muciniphila*, which erodes the mucous layer, leading to an abnormal inflammatory response. Given the mucinous degrading enzyme secreted by *Akkermansia*, it is hard to draw a conclusion whether *Akkermansia* aggravates or alleviates the injury caused by *C. malonaticus*-LPS, which is worthy of further study.

**Figure 10:**
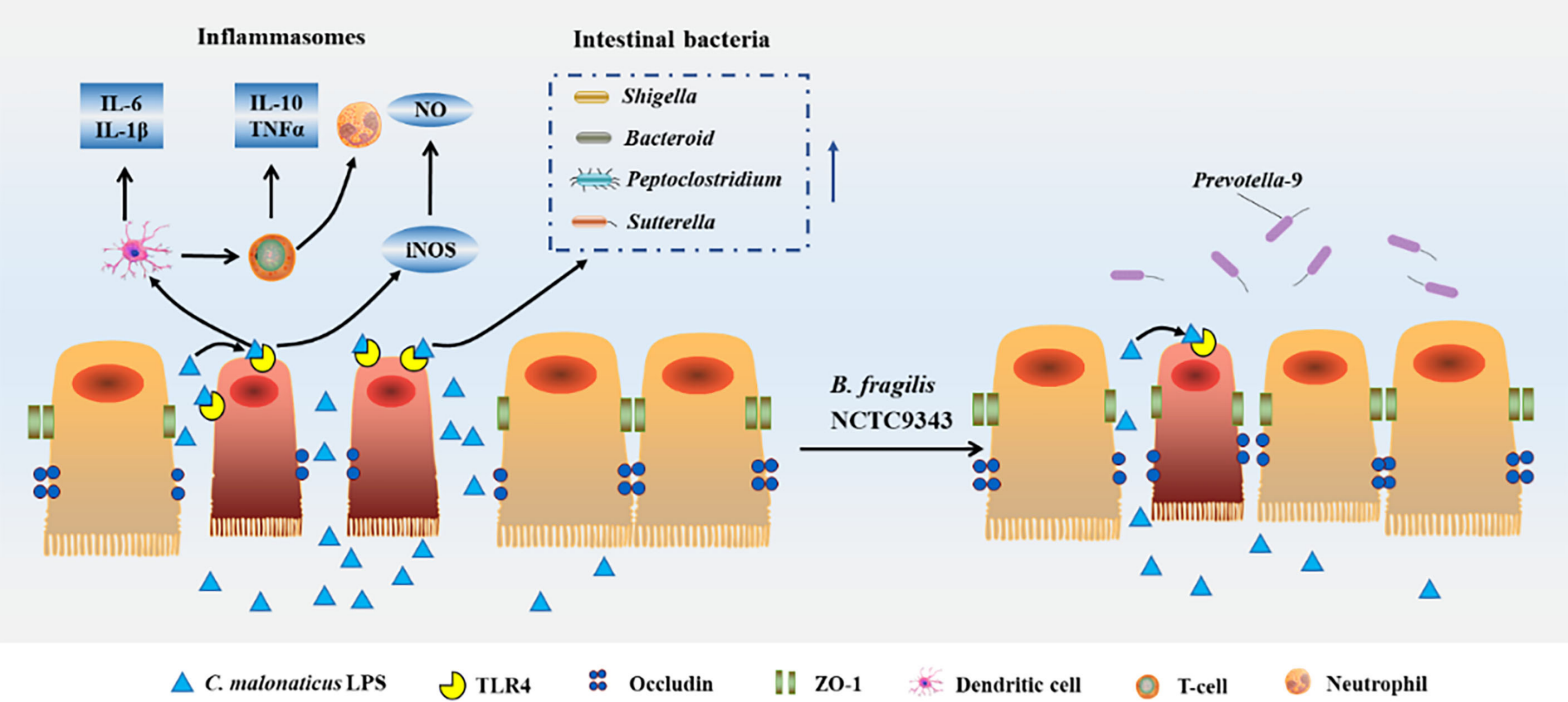
Host response caused by *Cronobacter*-LPS and reconstruction after B. *fragilis* NCTC9343 treatment.

Together, we demonstrated for the first time that *B. fragilis* NCTC9343 alleviates the deleterious effects of *C. malonaticus* LPS infection through four processes (**Figure 10**). First, *B. fragilis* might reduce the abundance of intestinal bacterial communities such as *Shigella, Peptoclostridium*, and *Sutterella*, which were induced by *C. malonaticus* LPS. *B. fragilis* might enhance gut microbiome like *Prevotella*-9 to recover *C. malonaticus* LPS-triggered flora disorder. Second, *B. fragilis* reversed the decreased expression of tight junction protein ccluding caused by *C. malonaticus* LPS. Additionally, *C. malonaticus* LPS increased the expression of TLR4, TNF-α, IL-Iβ, IL-6, IL-10, and iNOs, and those effects can be reversed by *B. fragilis* treatment. Finally, *B. fragilis* ameliorates intestinal injury caused by *C. malonaticus* LPS in animals.

## Data availability statement

The original contributions presented in the study are publicly available. Cronobacter malonaticus lipopolysaccharide-induced the intestinal microbiota data is available here: https://figshare.com/s/6ea48fef600d37ac2060, Doi: 10.6084/m9.figshare.19619721. Changes of intestinal microbiota when Bacteroides fragilis ameliorates Cronobacter malonaticus lipopolysaccharide-induced pathological injury data is available here: https://figshare.com/s/5b45646d0f6f1b23f390, 10.6084/m9.figshare.19624323. The raw data is available here: 10.6084/m9.figshare.19679511. For further inquiries you can contact the corresponding authors.

## Ethics statement

The animal study was reviewed and approved by Biomedical Ethics Committee, Hefei University of Technology.

## Author contributions

NL contributed to the data analysis, drafted the article, and revised the article. XZ contributed to the design of the experiments. SF contributed to the article revision. DZ, YS, JZ, YD, and JW were responsible for the data collection, analysis, and interpretation. QW and YY contributed to the conception and design of the experiments. All authors contributed to the article and approved the submitted version.

## Funding

We gratefully acknowledge the financial support of the National Natural Science Foundation of China (32102104; 31972175) and the Outstanding Youth Fund of Anhui Natural Science Foundation (2208085J11), the Fundamental Research Funds for the Central Universities (JZ2022HGTB0271), and the China Postdoctoral Science Foundation (2021M690854).

## Conflict of interest

The authors declare that the research was conducted in the absence of any commercial or financial relationships that could be construed as a potential conflict of interest.

## Publisher’s note

All claims expressed in this article are solely those of the authors and do not necessarily represent those of their affiliated organizations, or those of the publisher, the editors and the reviewers. Any product that may be evaluated in this article, or claim that may be made by its manufacturer, is not guaranteed or endorsed by the publisher.
